# Supplementary material for: Geographical variation and predictors of missing essential newborn care items during the immediate postpartum period in Ethiopia: Spatial and multilevel count analyses
Source: PLoS One. 2024 Sep 6;19(9):e0308104. doi: 10.1371/journal.pone.0308104 (PMC11379150; doi:10.1371/journal.pone.0308104)
Supplement: Attachment — Submitted filename: Response to reviewers.docx [file pone.0308104.s001.docx]

**A point-by-point response to editor and reviewers**

**Authors’ Response to Academic Editor**

**Dear: Amanuel Abajobir, PhD, Academic Editor, Plos One**

We thank you for a thorough reading and constructive comments and suggestions on our manuscript and for the opportunity to revise and resubmit. We are pleased to submit the revised version of the manuscript titled “**Geographical variation and predictors of missing essential newborn care during the immediate postpartum period in Ethiopia: Spatial and multilevel count analyses**” for your consideration in the special collection of **Plos One**. The comments of the editors and the reviewers were highly insightful and enabled us to greatly improve the quality of our manuscript. In this revised manuscript we made substantial changes to address your concerns in a point-by-point response. We appreciate your time and look forward to your response and we are very keen to incorporate further comments, if any, for the betterment of the final manuscript.

On the following pages, you will find our responses to the comments and suggestions raised by the esteemed editor and reviewer.

Sincerely,

Aklilu Habte (MPH)(corresponding author)

[**aklilihabte57@gmail.com**](mailto:aklilihabte57@gmail.com)

**Response to Journal requirements**

**1.** Please ensure that your manuscript meets PLOS ONE's style requirements, including those for file naming.

**Response:** we already prepared the manuscript as per the journal requirement and again we rechecked the compliance towards it during the submission of our revised manuscript.

**2.** In the online submission form, you indicated that [The data for this study were obtained from the DHS program with a reasonable request. Thus, the one who needs the data supporting the findings of this study can get it in anonymized form from the DHS website at https://www.dhsprogram.com upon reasonable request in the same manner as the authors did.].

All PLOS journals now require all data underlying the findings described in their manuscript to be freely available to other researchers, either 1. In a public repository, 2. Within the manuscript itself, or 3. Uploaded as supplementary information.

This policy applies to all data except where public deposition would breach compliance with the protocol approved by your research ethics board. If your data cannot be made publicly available for ethical or legal reasons (e.g., public availability would compromise patient privacy), please explain your reasons on resubmission and your exemption request will be escalated for approval.

**Response:** The DHS data policy doesn’t allow sharing of the dataset with third parties for other than initially registered purposes. Thus, we suggested the readers access the data on the above-mentioned link based on the reasonable request.

**3.** We note that Figure(s) [1,4,5,6,7,8 and 9,] in your submission contain [map/satellite] images which may be copyrighted. All PLOS content is published under the Creative Commons Attribution License (CC BY 4.0), which means that the manuscript, images, and Supporting Information files will be freely available online, and any third party is permitted to access, download, copy, distribute, and use these materials in any way, even commercially, with proper attribution. For these reasons, we cannot publish previously copyrighted maps or satellite images created using proprietary data, such as Google software (Google Maps, Street View, and Earth). For more information, see our copyright guidelines: http://journals.plos.org/plosone/s/licenses-and-copyright.We require you to either (1) present written permission from the copyright holder to publish these figures specifically under the CC BY 4.0 license, or (2) remove the figures from your submission:

1. You may seek permission from the original copyright holder of Figure(s) [1,4,5,6,7,8 and 9,] to publish the content specifically under the CC BY 4.0 license.

We recommend that you contact the original copyright holder with the Content Permission Form (http://journals.plos.org/plosone/s/file?id=7c09/content-permission-form.pdf) and the following text:

“I request permission for the open-access journal PLOS ONE to publish XXX under the Creative Commons Attribution License (CCAL) CC BY 4.0 (http://creativecommons.org/licenses/by/4.0/). Please be aware that this license allows unrestricted use and distribution, even commercially, by third parties. Please reply and provide explicit written permission to publish XXX under a CC BY license and complete the attached form.”

**Response:** We appreciate your concern to assure the ethical issues. However, all the aforementioned figures (1,4,5,6,7,8 and 9) in our manuscript are not copyrighted rather they are the result of spatial analysis that we have run in ArcGIS and SaTScan software. The GPS and DHS data that contain Shapefile and other relevant variables were obtained from the DHS office by explaining the objective of the study through online requests. Then, in order to get those figures, we import the relevant data extracted from the 2016 Ethiopian Demographic Health Survey reports and the shapefile of Ethiopia obtained from the 2016 Ethiopian Central Statistical Agency (CSA).To indicate this, we already cited the source of the shapefile alongside each figure. The shape file that we used to construct the figures can be accessed by one of the following links:

1. <https://data.humdata.org/dataset/cb58fa1f-687d-4cac-81a7-655ab1efb2d0>
2. <https://gadm.org/download_country.html>

Therefore, the maps presented in our study are not copyrighted rather they were the outputs of our spatial analysis results which are the result of those Shapefiles and projected CVS files in ArcGIS. This is the actual procedure that we employed in our present and earlier studies, as well as other Ethiopian researchers. Again we assure you that the figures presented in our study are not copyrighted but rather our spatial analysis results.

**Response to Additional Editor Comments:**

**General Comment:** Overall, the manuscript provides a comprehensive overview of the neonatal care landscape in Ethiopia, particularly focusing on the postnatal period and essential newborn care (ENC) services. Here's a breakdown of the review focusing on the quality of English, writing, and content:

**Response:** Thank you for your positive and constructive comments and suggestions we got all of them crucial in the improvement of the manuscript. Accordingly, we tried to respond to your possible suggestions as follows:

**Comment 1:** There are a few instances where sentences could be refined for clarity and flow. For instance, in the Methods and Materials section, sentences like "Respondents were selected using a stratified two-stage cluster sampling technique, having each region divided into urban and rural areas" could be rephrased for smoother reading, such as "Respondents were selected through a stratified two-stage cluster sampling technique, where each region was divided into urban and rural areas." Such minor adjustments can enhance readability without altering the technical content. Additionally, there are some instances of lengthy sentences and complex structures that could be simplified for better comprehension, especially for readers not familiar with the subject matter. Breaking down complex sentences into smaller, digestible parts can improve readability and understanding.

**Response:** Thank you for your insightful suggestions. We tried to correct some vague and complex sentences and highlighted them throughout the "Revised Manuscript with Track Changes"

**Comment 2:** Methods and Materials: Some parts could benefit from concise explanations to avoid overwhelming the reader with technical details. For example, the explanation of spatial autocorrelation and spatial interpolation could be simplified for clarity.

**Response:** Thank you for your suggestion. Initially, we did this intending to make the statements more clear to readers. Now, we entirely concur with your point of view because detailed explanations can be confusing to readers, thus we attempted to simplify certain difficult remarks.

Thank you for your constructive comments and suggestions, which we got as valuable input in the improvement of the manuscript. We received all of them as a valuable contribution to our ongoing work. In the following section, we tried to respond to all the possible comments and suggestions from reviewer #1

_____________________________END________________________________________

THANK YOU!!!

**Authors’ Response to Reviewer**#**1**

**General comment:** Demographic Health Survey is a well-written manuscript. Some comments to consider before publication are listed below.

**Response:** Dear Reviewer 1, thank you very much for taking the time to review our work and for your positive feedback. We received your thoughtful, and generous review, along with helpful feedback and suggestions, as a valuable contribution to our ongoing work. We have tried to address all the possible comments and suggestions raised by you in the following session.

**Comment 1:** Your abstract method section does not tell us which ANOVA you used

**Response:** thank you for your meticulous review. It was to mean a One-way ANOV and we have corrected and highlighted it in the ‘Abstract’ section of the "Revised Manuscript with Track Changes" Page 2, Line 50

**Comment 2:** Why SPSS 16, while you can do 25 or more what is the secret?

**Response:** It was not SPSS v16 it was STATA version 16 which was the most updated version at hand during the time of our analysis.

**Comment 3:** The abstract does not list what is the essential newborn care or defining variables

**Response:** Thank you for your thoughtful inquiry. As the abstract should be with a limited amount of words, initially we tried to make it short and precise. Now, as per your suggestion, we incorporated the items and highlighted them in the ‘Abstract’ section of the "Revised Manuscript with Track Changes" Page 2, Line 46-49

**Comment 4:** Abstract result focuses only on spatial results like there are no other variables

**Response:** We tried to incorporate the results of both spatial and multilevel analysis in a more short and precise way. For your clarity, the results other than spatial analysis were highlighted in the ‘Abstract’ section of the "Revised Manuscript with Track Changes" Page 2, Lines 58-63. **Comment 5:** Include the success number with percent in abstract and result sections …..(()60%)

**Response:** We have corrected and highlighted it in the ‘Abstract’ section of the "Revised Manuscript with Track Changes" Page 2, Line 56.

**Comment 6:** The level ENC is lower in your abstract compared to nothing……. Please avoid partial statements as this is a common problem throughout. For example, the government and policymakers should tailor strategies to hotspot areas by implementing activities and interventions…. What strategies…??? What implementation?? Just avoid general statements and non-practical recommendations or conclusions, this makes the study nonsense.

**Response:** Thank you for your inquiry and constructive suggestions. From the start, we concluded that Ethiopia had a higher level of missing ENC. The conclusion was reached after comparing and contrasting the current statistics with previous large-scale studies undertaken in Ethiopia and other countries. For that matter, we kindly ask you to look into the ‘Discussion’ section of the "Revised Manuscript with Track Changes" Page 21, Lines 454-457. Regarding our recommendation, it lacks specificity and thus we made an amendment and highlighted it in the ‘Abstract’ section of the "Revised Manuscript with Track Changes" Page 3, Lines 66-68

**Comment 7:** However, the drop in NNM has been more static, falling from 58 deaths per 1000 live births in 2000 to 29 deaths per 1000 births in 2016. This statement is not clear. I do know how something static could have this much variation. Please, follow the academic way of writing, your writing lacks details and makes partial statements and it is not to the point of focus. You need to go through the whole document and make clear sense of those limited ideas.

**Response:** It was to mean the figure was not satisfactory as per the government plan and to show it is vital to work on newborns. We have made amendments to the statement and highlighted it in the ‘Introduction’ section of the "Revised Manuscript with Track Changes" Page 3, Lines 81-82. We gave due emphasis to your comments and suggestions throughout the revised version of the manuscript and we have highlighted them.

**Comment 8:** Is PPP just 42 days? Exactly what information is necessary for a mother during postpartum?

**Response:** Yes, the postpartum period covers from the time of delivery to 42 days postpartum. However, our study mainly focuses on the contents of care provided during the immediate postpartum period (i.e. within 2 days following birth) which is the focus of DHS data. In addition, other preventive and curative services could be provided during PPP but they were not our current study’s interest.

**Comment 9:** intrapartum and postpartum care remained mostly responsible for a steady reduction in neonatal death in Ethiopia……what are those intrapartum and postpartum care missing in Ethiopia?

**Response:** Thank you for asking. The main intrapartum care was skilled delivery service and the postpartum one was essential newborn care services. We made slight amendments to the statement and highlighted it in the ‘Introduction’ section of the "Revised Manuscript with Track Changes" Page 3, Lines 104-106.

Comment 10: Your finding indicates 60% of ENC is missing, but your literature does not have any information related to this excepting million global deaths. You need to reconstruct your introduction according to your main objective.

**Response:** Thank you for your suggestion. Accordingly, we added some figures regarding the level of missing ENC from previously conducted large-scale studies. That statement was highlighted in the ‘Introduction’ section of the "Revised Manuscript with Track Changes" Page 3, Lines 101-104.

**Comment 10:** Based on this evidence the gap for which you conducting this study is clearly identified and you need to deal with more.

**Response:** thank you for your suggestion to strengthen the real gaps that were addressed by this study. Accordingly, we highlighted the gaps that were addressed by the current study in the last paragraph of the ‘Introduction’ section of the "Revised Manuscript with Track Changes" Page 4.

**Comment 11:** Have clear data preparation and how you handled missing or if other difficulties exist as this data has many gaps

**Response:** we have removed those respondents without any information on the uptake of PNC within 42 days. We have mentioned this in the ‘Population of the study’ section of the "Revised Manuscript with Track Changes" Page 5, Lines 138-142. Beyond that, we didn’t face any difficulty with missing values on other important covariates.

**Comment 12:** Provide a clear procedure for your analysis and assumptions for low scholars' understanding. You have that for spatial analysis but not for others

**Response:** Thank you for your insightful suggestion. We have mentioned the details of assumptions that we followed while running a generalized linear model (a multilevel negative binomial regression) in the ‘Data management and statistical analysis’ section of the "Revised Manuscript with Track Changes" Page 10, Lines 257-264.

**Comment 13:** How do you apply OLS with dichotomous data?

**Response:** The ordinary least square(OLS) that we applied in the current study was from the spatial regression dimension, not from the perspective of ordinary linear regression. we kindly ask you to look into the details of the spatial regression section.

**Comment 14:** You have very large result presentations, you may need to make a summary of results at the end

**Response:** thank you for your suggestion. However, the journal requirement doesn’t allow us to add the summary part to the result section. The only section to summarize our findings was the ‘Abstract’ section and we tried to summarize all the main findings here. By emphasizing your remarks, we attempted to make the result section more concise and precise by trimming some of the lengthy phrases.

**Comment 15:** Somalia, Afar, and Gambella are usually underperforming in most studies what is different about them?

**Response:** thank you for asking. These regions are known for low maternal, neonatal, and child health service uptake due to a variety of reasons. To begin, as compared to other regions, those regions, particularly Afar and Somalia, are known to have a shortage of healthcare facilities, limited healthcare providers, and a lack of medical supplies and equipment to provide ENC services. In this specific scenario, we tried to mention the possible justifications behind the high proportion of women missing ENC in the second paragraph of the discussion section.

**Comment 16:** Result comparison could make more sense if you can make a more focused comparison with similar setups and reasonably compare how that is common in most developing countries. Something is missing in your initial paragraph – focus

**Response:** we appreciate your insightful inquiry. We entirely agree with your suggestion. Accordingly, we tried to make a comparison with studies conducted in developing countries and list out the possible justifications. We have highlighted the corrected statement in the ‘Discussion’ section of the "Revised Manuscript with Track Changes" Page 19, Lines 455-460.

**Comment 17:** I already commented on how to make the conclusion more practical earlier, thus, apply it here.

**Response:** We agree with your idea and now we have tried to make the conclusion more practical and highlighted it in the ‘Conclusion’ section of the "Revised Manuscript with Track Changes" Page 24, Lines 543-546.

Thank you for your constructive comments and suggestions, which we got as valuable input in the improvement of our manuscript. We received all of them as a valuable contribution to our ongoing work.

**_________________________________________________________________________________________________________END_______________________________________**

**THANK YOU!!!**
