# Supplementary material for: Geographical variation and predictors of missing essential newborn care items during the immediate postpartum period in Ethiopia: Spatial and multilevel count analyses
Source: PLoS One. 2024 Sep 6;19(9):e0308104. doi: 10.1371/journal.pone.0308104 (PMC11379150; doi:10.1371/journal.pone.0308104)
Supplement: Attachment — Submitted filename: Response to editor.docx [file pone.0308104.s002.docx]

**A point-by-point response to editor and reviewers**

**Authors’ Response to Academic Editor**

**Dear: Amanuel Abajobir, PhD, Academic Editor, Plos One**

We thank you for a thorough reading and constructive comments and suggestions on our manuscript and for the opportunity to revise and resubmit. We are pleased to submit the revised version of the manuscript titled “**Geographical variation and predictors of missing essential newborn care during the immediate postpartum period in Ethiopia: Spatial and multilevel count analyses**” for your consideration in the special collection of **Plos One**. The comments of the editors and the reviewers were highly insightful and enabled us to greatly improve the quality of our manuscript. In this revised manuscript we made substantial changes to address your concerns in a point-by-point response. We appreciate your time and look forward to your response and we are very keen to incorporate further comments, if any, for the betterment of the final manuscript.

Sincerely,

Aklilu Habte (MPH)(corresponding author)

[**aklilihabte57@gmail.com**](mailto:aklilihabte57@gmail.com)

**Response to Journal requirements**

**1.** Please review your reference list to ensure that it is complete and correct. If you have cited papers that have been retracted, please include the rationale for doing so in the manuscript text, or remove these references and replace them with relevant current references. Any changes to the reference list should be mentioned in the rebuttal letter that accompanies your revised manuscript. If you need to cite a retracted article, indicate the article’s retracted status in the References list and also include a citation and full reference for the retraction notice.

**Response:** Thank you for your reminder to look into the appropriateness of the references that we used for this work. Accordingly, we got all the references were relevant and none of them were retracted.

**Response to Additional Editor Comments:**

**Comment 1:** Although the revised version has addressed most of the comments, significant language edits are still required to meet the standards of scientific writing before it can be formally accepted for publication.

**Response:** Thank you for acknowledging that we addressed most of the comments raised by the reviewers and editor. As per your suggestion, we tried to give due emphasis to the grammatical and typological issues throughout the manuscript and we tried to correct and highlight them throughout the "Revised Manuscript with Track Changes"

**Comment 2:** While revising your submission, please upload your figure files to the Preflight Analysis and Conversion Engine (PACE) digital diagnostic tool, <https://pacev2.apexcovantage.com/>. PACE helps ensure that figures meet PLOS requirements. To use PACE, you must first register as a user. Registration is free. Then, login and navigate to the UPLOAD tab, where you will find detailed instructions on how to use the tool. If you encounter any issues or have any questions when using PACE, please email PLOS at [figures@plos.org](mailto:figures@plos.org) Please note that Supporting Information files do not need this step.

Response: Thank you for your suggestion. However, we initially submitted the figures as per the journal’s requirement and we want to assure you that they were PACE corrected figures.

Thank you for your constructive comments and suggestions, which we got as valuable input in the improvement of the manuscript. We received all of them as a valuable contribution to our ongoing work.

_____________________________END________________________________________

THANK YOU!!!
