# Supplementary material for: Geographical variation and predictors of missing essential newborn care items during the immediate postpartum period in Ethiopia: Spatial and multilevel count analyses
Source: PLoS One. 2024 Sep 6;19(9):e0308104. doi: 10.1371/journal.pone.0308104 (PMC11379150; doi:10.1371/journal.pone.0308104)
Supplement: Attachment — Submitted filename: Response to reviewers.docx [file pone.0308104.s003.docx]

**A point-by-point response to editor and reviewers**

**Authors’ Response to Academic Editor**

**Dear: Amanuel Abajobir, PhD, Academic Editor, Plos One**

We thank you for a thorough reading and constructive comments and suggestions on our manuscript and for the opportunity to revise and resubmit. We are pleased to submit the revised version of the manuscript titled “**Geographical variation and predictors of missing essential newborn care during the immediate postpartum period in Ethiopia: Spatial and multilevel count analyses**” for your consideration in the special collection of **Plos One**. The comments of the editors and the reviewers were highly insightful and enabled us to greatly improve the quality of our manuscript. In this revised manuscript we made substantial changes to address your concerns in a point-by-point response. We appreciate your time and look forward to your response and we are very keen to incorporate further comments, if any, for the betterment of the final manuscript.

On the following pages, you will find our responses to the comments and suggestions raised by the esteemed editor and reviewer.

Sincerely,

Aklilu Habte (MPH)(corresponding author)

[**aklilihabte57@gmail.com**](mailto:aklilihabte57@gmail.com)

**Response to editors’ comment**

**1.** Please remove any reference to any 'ethnicity' in the 'Classification' of the submission system.

**Response:** thank you so much for your suggestion. Accordingly, we removed it in the submission system during our submission to revised version of the manuscript.

_____________________________END________________________________________

THANK YOU!!!

**Authors’ Response to Reviewer**#**2**

**Comment 1:** The manuscript requires major editorial editing throughout. The cited references, abbreviations etc need to be spaced out from the words and figures consistently across the entire manuscript, including the tables. Please refer to a PLOS journal article for an example.

**Response:** Dear Reviewer 2, thank you very much for taking the time to review our work and for your positive feedback. We received your thoughtful, and generous review, along with helpful feedback and suggestions, and we have corrected all the raised editorial issues throughout the revised version of the manuscript. For your convenience, we made a highlight to show the space between the reference and words.

**Comment 2:** The analysis conducted is comprehensive, but the manuscript could be further improved.

**Response:** we appreciate your concerns. However, we assure you that the whole content of the manuscript was the mirror image of the analysis. We performed descriptive, multilevel, and spatial analyses and all results were well presented accordingly.

**Comment 3:** Line 175, full name for the abbreviation for BPCR.

**Response:** Thank you very much for your meticulous review. It was a typo error and we have corrected and highlighted it in the ‘Measurement of variables of the study’ section of the "Revised Manuscript with Track Changes" on page 6, Lines 174-175

**Comment 4:** Line 267, Line 271, Line 315, a one or two-tailed test is to be stated.

**Response:** we appreciate your insightful inquiry. We performed an independent t-test and one-way Analysis of variance (ANOVA) and we have corrected and highlighted it in the ‘data analysis’ section of the "Revised Manuscript with Track Changes" on page 10, Line 267

**Comment 5:** Line 280, the sentence for Var(w) is to be revised. Var (w) refers to the variance of the standard logistic distribution used in the logistic link function.

**Response:** thank you for your meticulous review and insightful suggestion. We have corrected and highlighted it in the ‘Data analysis’ section of the "Revised Manuscript with Track Changes" on page 10, Lines 280-281

**Comment 6:** Line 284, the PCV range and interpretation to be provided e.g. 0 < PCV < 1.

**Response:** thank you for your suggestion. We have added and highlighted it in the revised version of the manuscript, Lines 285-286, Page 10

**Comment 7:** Line 286, more information for MOR to be provided e.g. quantify the unexplained cluster-level heterogeneity.

**Response:** we have added the statement and highlighted it in the revised version of the manuscript, Lines 287-288, Page 10.

**Comment 8:** Line 369, typo p<000

**Response:** We have corrected and highlighted it on Line 371, Page 15

**Comment 9:** Line 379, Line 391, Line 393, Line 396, R2, R squared, R-square is to be written as R^2 or R with 2 as superscript or R-squared. Need to be consistent/standardized.

**Response:** Thank you for your meticulous review. We have corrected it as R^2^ and highlighted it as per your suggestion.

Comment 10: Table 6 current age, the values o.83 and o.93 to be revised as 0.83 and 0.93 respectively.

**Response:** we have corrected and highlighted it in Table 6 of the "Revised Manuscript with Track Changes" Page 19.

**Comment 11:** References did not conform to the journal format.

**Response:** Thank you so much for your suggestion. As the Plos One journal requirement is the Vancouver style we made citations accordingly. Beyond that, this is the reference style that we used during our prior publication experience at this journal.

Thank you for your constructive comments and suggestions, which we got as valuable input in the improvement of our manuscript. We received all of them as a valuable contribution to our ongoing work.

**_________________________________________________________________________________________________________END_______________________________________**

**THANK YOU!!!**
